# Numerical analysis and optimization of continuous texture geometries in thermo-hydrodynamic journal bearings

**DOI:** 10.1038/s41598-025-28064-9

**Published:** 2025-12-04

**Authors:** Omar A. Keshk, Hassan ELGamal, Ossama Mokhiamar, Ahmed M. Saleh

**Affiliations:** 1 Mechatronics Engineering Department, Alexandria Higher Institute of Engineering and Technology, Smouha, Alexandria, Egypt; 2 Mechanical Engineering Department, Faculty of Engineering, Alexandria University, El-Chatby, Alexandria, 21544 Egypt

**Keywords:** Journal bearings, Surface texturing, Texture optimization, Monte Carlo method, Load capacity, Mechanical engineering, Theory and computation

## Abstract

Journal bearings play a critical role in many engineering systems, where their performance directly affects operational efficiency and reliability. This study investigates the effect of surface texturing on the thermo-hydrodynamic (THD) behavior of journal bearings with the goal of enhancing load-carrying capacity (LCC). Departing from conventional dimple-based textures, five continuous texture shapes are introduced and systematically analyzed using COMSOL Multiphysics. The thermo-hydrodynamic model is validated against the experimental data of Ferron, J., Frene, J. & Boncompain, R. A study of the thermohydrodynamic performance of a plain journal bearing comparison between theory and experiments. (1983). Optimization of texture parameters including height and distribution in both axial and circumferential directions is performed numerically using the Monte Carlo method. The study also evaluates the impact of texture placement in three distinct regions of the bearing surface. Results show that optimized continuous textures significantly outperform smooth bearings, achieving up to 144% increase in LCC. These findings offer new insights into texture design for high-performance journal bearings under thermal and hydrodynamic constraints.

## Introduction

Journal bearings are essential components in a wide range of mechanical systems, providing crucial support for rotating shafts and minimizing friction between moving parts. Their performance and reliability are integral to the efficiency and longevity of machinery, particularly in high-load and high-speed applications. Traditionally, journal bearings operate on the principle of hydrodynamic lubrication, where a thin film of lubricant separates the bearing surfaces, reducing wear and energy loss^2–5^. However, the increasing industrial demands for improved performance, efficiency, and durability have driven significant innovations, particularly around surface texturing^6–8^

Surface texturing, involving the creation of micro- or nano-scale patterns on the bearing surface, has emerged as a powerful technique to enhance the tribological properties of journal bearings^9^. Numerous studies have shown that optimized surface textures can increase LCC, reduce friction, and enhance wear resistance^5,10–12^. For example, Saleh et al.^13^ demonstrated that specific texture patterns can significantly improve bearing performance under various operational conditions. Kumar and Pandey^4^ provided a comprehensive review of recent advances in surface texturing, highlighting its potential to alter surface microgeometry in ways that optimize lubrication and reduce wear. Similarly, Wang Y et al.^14^ emphasized the role of texture design in modifying the lubrication regime, which directly influences the overall efficiency of journal bearings.

Thermal management is another critical aspect of journal bearing performance, significantly influenced by surface texturing^15^. Filgueira et al.^16^ explored the thermal and hydrodynamic effects of various texture shapes and distributions, finding that optimized textures can manage temperature distribution within the lubricant film, thereby improving bearing stability and longevity. Mishra and Aggarwal^5^ further discussed the impact of strategically placed textures on mitigating thermal gradients, which can prevent localized overheating and extend the bearing’s operational life. Additionally, Lu and Khonsari^6^ and Cupillard et al.^17^ highlighted the importance of texture orientation in optimizing thermal management and load distribution, demonstrating that specific orientations can significantly enhance the bearing’s performance under thermal stress.

Despite considerable progress in surface texturing, several critical gaps remain. Most previous studies have focused on discrete texture shapes such as dimples and grooves, which, while effective in some cases, often suffer from reduced performance under varying operating conditions and present manufacturing challenges^2,6,18,19^. Continuous texture shapes defined by smooth functional variations have been suggested as a promising alternative, offering better film continuity and pressure recovery^2,4,6^ yet remain underexplored, particularly under thermo-hydrodynamic conditions. Furthermore, partial texturing has been shown to be more beneficial than full-surface designs, especially in high-load regions, as noted by Brizmer et al.^8^ and Cupillard et al.^17^. However, comprehensive studies that combine continuous texture modeling with systematic optimization techniques are limited. Recent advances have further emphasized this trend. For instance, Kong et al.^20^ conducted topology optimization of textured journal bearings, highlighting the influence of geometric configuration on load-carrying capacity. Lu et al.^21^ analyzed texture performance under varying lubrication states, while Muchammad et al.^22^ investigated multistep and partial texturing effects in elastohydrodynamic regimes. These studies reaffirm the critical role of geometry and placement optimization in enhancing bearing performance. In particular, the effects of texture height, density, and placement on performance indicators such as LCC, friction, and temperature distribution require further investigation^23–26^.

Unlike traditional surface texturing approaches that rely on discrete features such as dimples, grooves, or cavities, the continuous texture geometries proposed in this study are defined by smooth, uninterrupted mathematical functions (e.g., sinusoidal, or cosine-based waveforms). These geometries eliminate sharp edges and discontinuities in the lubricant film, which are often associated with turbulence, cavitation initiation, and localized pressure losses in discrete texture designs. The smooth variation of the surface profile in continuous textures promotes more uniform film pressure distribution, reduces thermal hotspots, and improves lubricant stability. Moreover, continuous textures can be tailored more flexibly to align with the hydrodynamic load zone, making them suitable for applications requiring both high performance and predictable wear behavior. To the authors’ knowledge, this study represents one of the first detailed numerical investigations comparing multiple continuous texture shapes in a fully thermo-hydrodynamic framework.

Optimization of texture geometry and operating conditions is crucial for maximizing the benefits of surface texturing. Lampaert et al.^27^ demonstrated that advanced numerical simulations are essential for optimizing surface textures under varying thermal conditions, showing that even minor adjustments in texture geometry can significantly impact bearing performance. Mishra and Aggarwal^5^, along with Kumar and Pandey^4^, highlighted the limitations of existing models in capturing the dynamic interactions between textured surfaces and lubricants, particularly under transient conditions. The importance of using sophisticated simulation tools to accurately predict the effects of texture placement and geometry on bearing performance was further stressed by Wang^28^ and Filgueira et al.^16^.

Recent advancements in hydrodynamic bearing research have introduced hybrid approaches to address thermal and mechanical performance limitations, particularly in high-speed, high-load applications. For instance, Bhat et al.^29^ proposed a novel thrust bearing pad design that integrates embedded cooling circuitry, deep recesses, and surface texturing, which led to a reduction in maximum pad temperature from 84 °C to 66 °C and an increase in peak pressure from 5.8 to 6.8 MPa. Similarly, in a study combining nanofluid lubrication with water-cooled bearing pads, thermal deformation was reduced from 36.5 to 22.3 µm, highlighting the benefits of synergistic cooling and lubrication enhancements^30^. Another recent work focused on optimizing lubricant flow and heat dissipation using CFD-based analysis of hybrid pad configurations, demonstrating a temperature drop from 85°deC to 71 °C and improved thermal reliability^31^. These studies underscore the importance of integrating thermal management strategies with surface design optimization an approach that complements the present work’s objective of improving load-carrying capacity and thermal stability through continuous surface texturing.

The present study builds upon and significantly extends the work of Masmoudi et al.^32^ and Saleh et al.^13^ in several key areas. While Masmoudi et al. focused on rectangular dimples with micro-textured roughness and used a hybrid CFD approach for steady-state performance evaluation, their texture distribution was limited to fixed geometries without full parametric optimization. Their findings, although insightful, did not explore continuous variation in the patterned surface elevation (texture profiles) or fully couple the thermal effects with optimization. Similarly, Saleh et al. employed finite difference modeling to evaluate multiple discrete convex and concave dimple shapes, concluding that curvature orientation significantly affects LCC. However, their work was restricted to evaluating pre-defined dimple shapes without exploring continuous mathematical texture functions or using optimization techniques.

In contrast, the present research introduces five mathematically defined continuous textures that allow smoother transitions in film thickness and pressure gradients. These textures are not limited to predefined dimples but are instead controlled by continuous sinusoidal surface functions. More importantly, this study applies a Monte Carlo optimization technique to determine the optimal combination of texture parameters (height, axial and circumferential distribution), which neither of the referenced studies pursued. The methodology also incorporates fully coupled thermo-hydrodynamic modeling using COMSOL Multiphysics, providing more accurate predictions of both pressure and thermal profiles.

To address the identified research gaps, this study optimizes continuous texture shapes for thermo-hydrodynamic (THD) journal bearings using advanced numerical techniques. It introduces, for the first time, a systematic numerical investigation of five mathematically defined continuous texture geometries: sine, cosine, absolute sine, exponential, and hybrid cosine—sine profiles optimized through a Monte Carlo approach. Unlike previous research focused on discrete dimples or grooves, the present work employs smooth, continuous surface functions to achieve stable film formation and improved thermal performance. The integration of full THD coupling within COMSOL Multiphysics, combined with large-scale Monte Carlo–based optimization (1000 iterations), establishes a new framework for parameter exploration in textured bearing design. The study further evaluates partial versus full texture placement and quantitatively demonstrates a 144% increase in load-carrying capacity compared to a smooth bearing, representing a significant step toward predictive, optimization-driven design of continuous textured surfaces in journal bearings.

The key objectives of this research are as follows:

### Optimization of Continuous Texture Shapes

This study explores five different continuous texture shapes sine-based, cosine-based, absolute sine, exponential, and cosine–sine hybrid profiles shown in Fig. 7 moving beyond traditional discrete dimples to enhance the hydrodynamic performance of journal bearings. The optimization aims to maximize LCC and reduce friction losses.

### Application of Monte Carlo technique

A systematic numerical approach, including the Monte Carlo optimization technique, is utilized to efficiently explore a wide range of texture configurations. This method enables the identification of optimal texture parameters that enhance bearing performance under varying operational conditions.

### Strategic texture placement

The study focuses on positioning surface textures in high-load regions of the bearing surface (Locations 1, 2, and 3, as illustrated in Fig. 5) to achieve maximum pressure distribution benefits. This strategic placement aims to improve lubricant film stability and extend the operational lifespan of the bearing system.

### Improvement of tribological performance

By refining texture parameters such as height, density, and spatial distribution, this research seeks to overcome the limitations observed in prior studies. The goal is to minimize frictional losses while maintaining effective thermal management within the lubricant film.

### Contribution to surface-textured bearing design

The insights obtained from this study provide a deeper understanding of the influence of continuous texture shapes on journal bearing performance. The findings contribute to the advancement of design methodologies for surface-textured bearings, offering new guidelines for optimizing tribological performance in practical engineering applications.

This research bridges the existing knowledge gap by integrating optimization techniques with a detailed numerical analysis of texture shape effects, leading to enhanced efficiency and reliability in journal bearings.

The physical model was first validated against the well-established experimental study by Ferron et al.^1^, a benchmark in thermo-hydrodynamic journal bearing research. Building on this validated model, five continuous texture shapes were designed and implemented, with their performance compared against discrete dimple textures as outlined by Masmoudi^32^. The findings demonstrate superior performance of continuous textures, particularly in enhancing LCC and managing thermal effects.

All simulations and optimizations were conducted using COMSOL Multiphysics, a robust tool for modeling the thermo-hydrodynamic behavior of journal bearings. This work presents the details of the approach, the results of the comparative study, and the optimization process, offering new insights into the design and application of surface-textured journal bearings for enhanced performance.

## Methodology

### Governing equations

The behavior of the thermo-hydrodynamic journal bearing system is governed by a combination of fluid flow, heat transfer, and thermal effects within the lubricant film and bearing materials. The simulation includes contributions from thin film flow, heat transfer within the lubricant film, heat transfer within the solid components, and heat exchange with the ambient environment. The following governing equations describe these phenomena:

#### Thin film flow (Reynolds equation)

The thin film flow within the journal bearing is modeled using the Reynolds equation, which describes the pressure distribution in the lubricant film. This equation assumes that the flow is laminar and that the film thickness (the local distance between the journal and bearing surfaces, which governs the formation of the hydrodynamic pressure field) is small compared to the bearing dimensions. The governing equation for pressure distribution is:

1 $$\frac{\partial }{\partial x}\left(\frac{{h}^{3}}{12\mu }\frac{\partial p}{\partial x}\right)+\frac{\partial }{\partial y}\left(\frac{{h}^{3}}{12\mu }\frac{\partial p}{\partial y}\right)=\frac{\partial h}{\partial t}+{u}_{0}\frac{\partial h}{\partial z}$$

This equation governs the pressure gradient and the resulting LCC of the lubricant film between the shaft and the bearing surface.

#### Heat transfer in the lubricant film (convection-conduction equation)

Heat transfer within the thin lubricant film is governed by the energy equation, which accounts for both conduction and convection effects. The temperature distribution within the lubricant film is affected by the viscous dissipation of mechanical energy into heat due to shear stresses, modeled as:

2 $$\rho_{f} C_{p,f} \left( {\frac{{\partial T_{f} }}{\partial t} + u \cdot \nabla T_{f} } \right) = k_{f} \nabla^{2} T_{f} + \mu \left( {\frac{\partial u}{{\partial y}}} \right)^{2}$$

$\left(\frac{\partial u}{\partial y}\right)$ represents the velocity gradient due to shear stresses within the film.

The right-hand side of the equation includes a viscous dissipation term $\mu {\left(\frac{\partial u}{\partial y}\right)}^{2}$, which accounts for the heat generated due to the shearing of the lubricant as it flows between the surfaces.

#### Heat transfer in solids

The heat conduction within the solid components of the journal bearing (such as the shaft and bearing) is described by Fourier’s law of heat conduction:

3 $${\rho }_{s}{C}_{p,s}\frac{\partial {T}_{s}}{\partial t}={k}_{s}{\nabla }^{2}{T}_{s}+{q}_{gen}$$

This equation governs the temperature distribution within the solid components (shaft and bearing) as they conduct heat away from the lubricant film and dissipate it to the surrounding environment.

#### Convective heat transfer (Newton’s law of cooling)

Heat loss to the ambient environment from both the shaft and the bearing surfaces occurs via convection. The convective heat transfer is described by Newton’s law of cooling:

4 $${q}_{conv}={h}_{c}\left({T}_{s}-{T}_{\infty }\right)$$

#### Coupling between fluid flow and heat transfer

The heat generated in the lubricant film due to viscous dissipation and the heat transfer between the solid components and the surrounding air are strongly coupled. The lubricant viscosity **μ** is temperature-dependent, and this coupling is expressed as:

5 $$\mu \left(T\right)={\mu }_{0}\left(\frac{b}{T}\right)$$

As the temperature in the lubricant film rises due to viscous dissipation, the viscosity decreases, affecting the pressure distribution and the LCC of the journal bearing.

#### Film thickness equation

The film thickness equation describes the gap between the bearing and the journal (shaft). The local film thickness h (x, y, t) is a function of the journal’s eccentricity and its rotational position. In cylindrical coordinates, for a journal bearing, the film thickness **h** is expressed as:

6 $$h\left( \theta \right) = c\left[ {1 + \varepsilon \cos \theta } \right] - h_{text}$$

This equation is used to determine the local film thickness for smooth (${\text{h}}_{\text{text}}=0$) or textured surface at any given angular position around the bearing surface, accounting for the eccentricity of the journal within the bearing housing.

**Table 1:** Operating conditions.

Journal radius	R_j_ = 50mm
External bearing radius	R_b_ = 100mm
Bearing length	L = 80mm
Radial clearance	C = 145μm
Lubricant viscosity at 40·°C	μ_0_ = 0.0277 Pa. s
Lubricant density at 40·°C	P = 860 kg/m^3^
Lubricant specific heat	C_o_ = 2000 J/kg °C
Lubricant thermal conductivity	K_o_ = 0.13 W/m·°C
Air thermal conductivity	K_a_ = 0.025 W/m·°C
Bush thermal conductivity	K_b_ = 250W/m °C
Shaft thermal conductivity	K_s_ = 50W/m·°C
Convection heat transfer coefficient	h_b_ = 80W/m^2^ °C
Inlet lubricant temperature	T_i_ = 40·C
Ambient temperature	T_a_ = 40·C
Inlet lubricant pressure	P_i_ = 70 k Pa

### Model validation

The thermo-hydrodynamic journal bearing model was first validated against the experimental results obtained by Ferron et al.^1^. This foundational study serves as a benchmark for assessing the accuracy of numerical predictions, particularly for key parameters such as maximum pressure and film temperature.

**Fig. 1:**
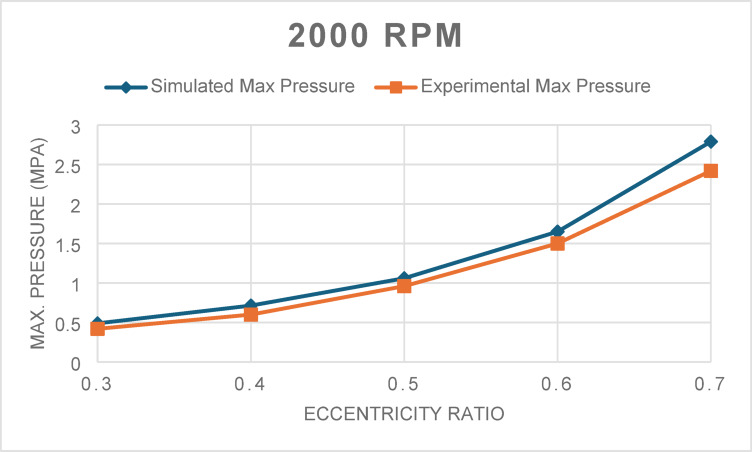
Maximum pressure for 2000 rpm at different eccentricity ratios.

**Fig. 2:**
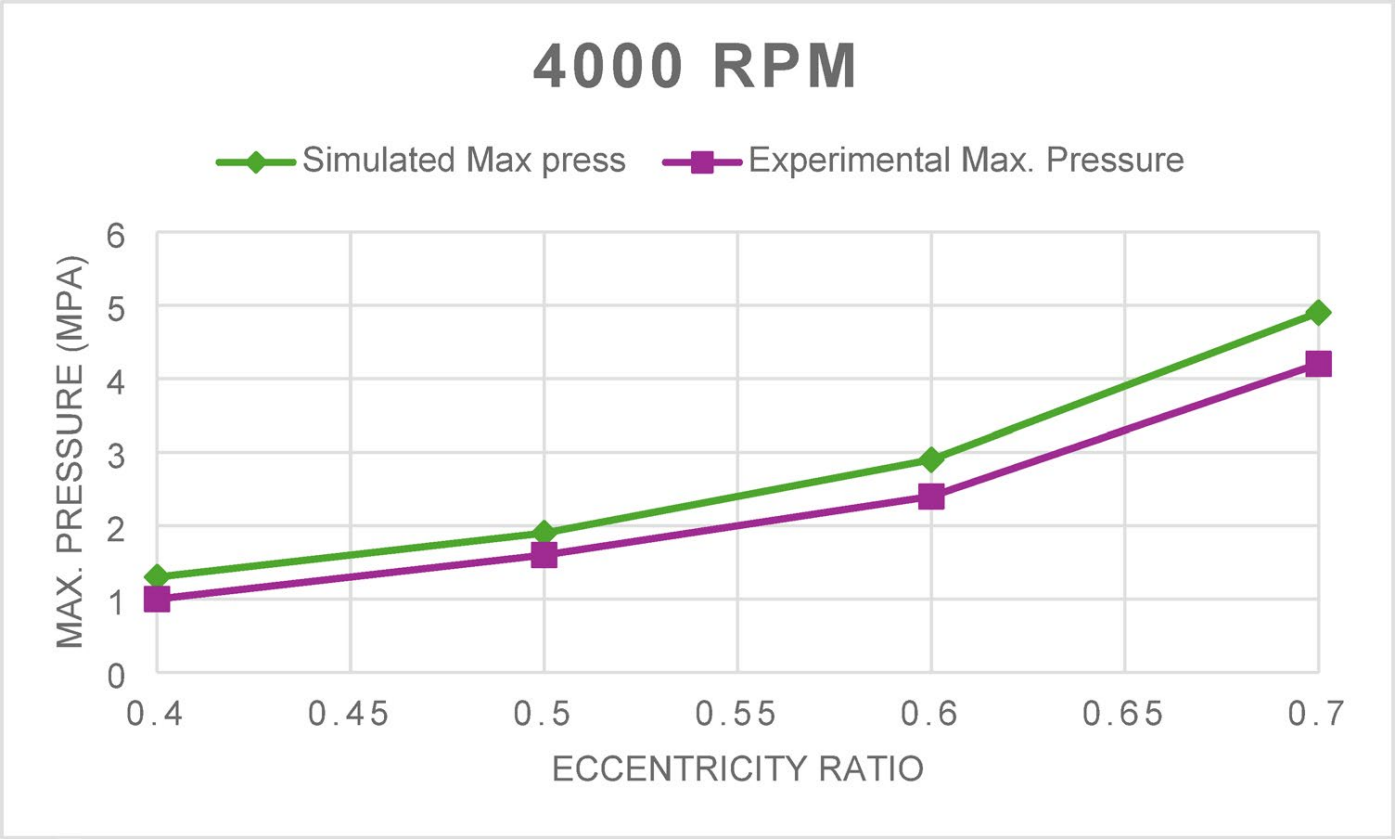
Maximum pressure for 4000 rpm at different eccentricity ratios.

The first validation step involved comparing the simulated maximum pressure with experimental data at rotational speeds of 2000 and 4000 rpm across eccentricity ratios ranging from 0.3 to 0.7. As shown in Figs. 1 and 2, the simulation results align well with the experimental values. The RMS error for 2000 rpm was 0.199 MPa, with a R^2^ value of 0.918, indicating strong agreement. For 4000 rpm, the RMS error was 0.480 MPa with an R^2^ of 0.818, confirming good model fidelity even at higher speeds.

**Fig. 3:**
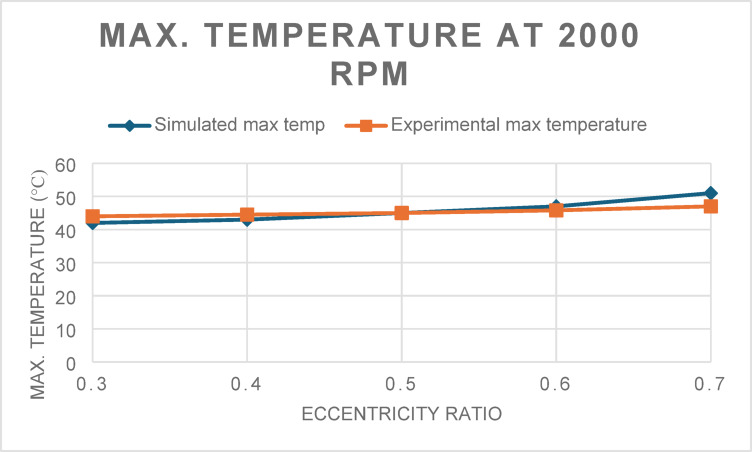
Maximum Temperature for 2000 rpm at different eccentricity ratios.

**Fig. 4:**
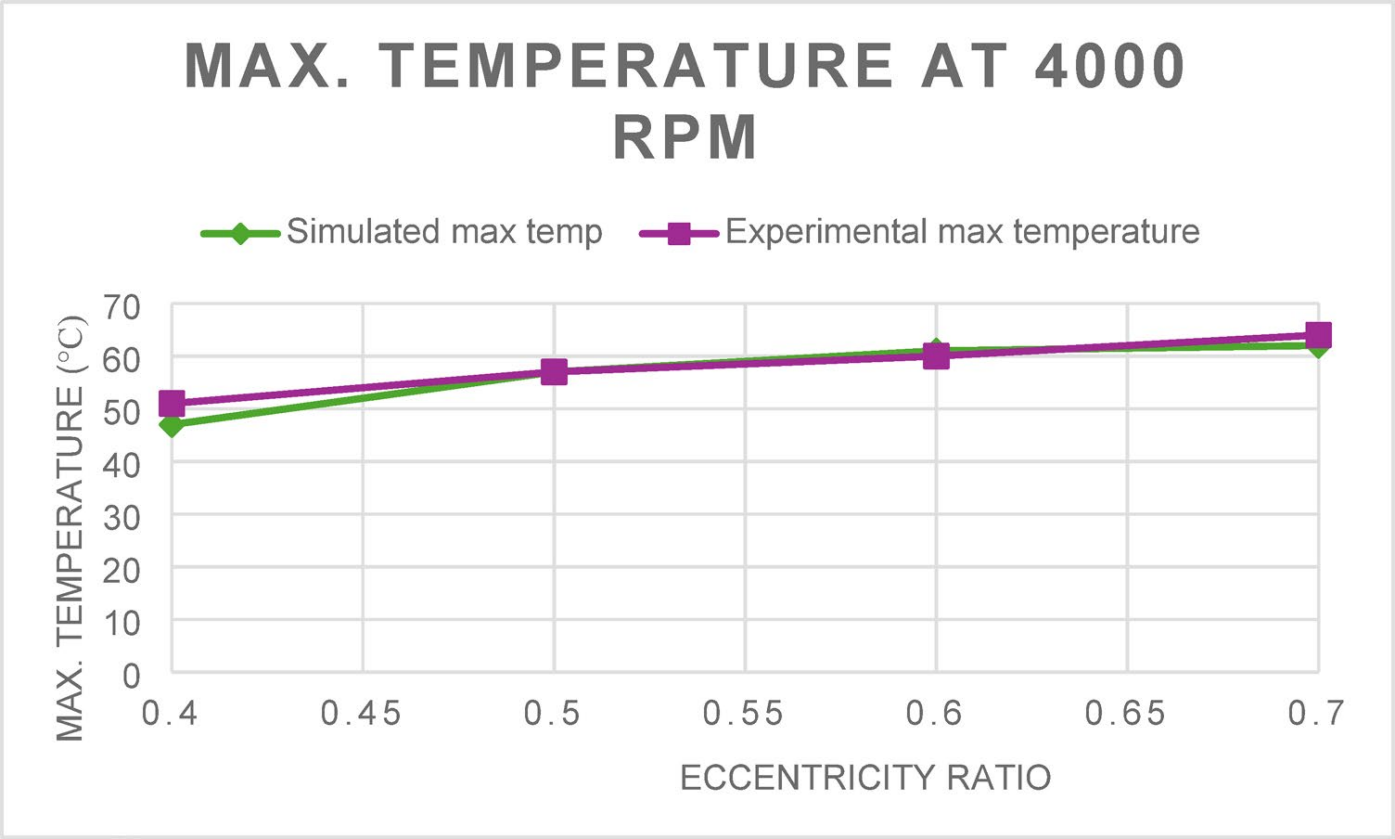
Maximum Temperature for 2000 rpm at different eccentricity ratios.

Similarly, the simulated maximum lubricant film temperatures were validated against experimental measurements (Figs. 3 and 4). At 2000 rpm, the RMS error was 1.99 °C, though the R^2^ value was –1.63, suggesting a weaker correlation at lower speeds due to minor fluctuations in thermal boundary assumptions. For the 4000-rpm case, the model yielded a more consistent prediction with an RMS error of 2.29 °C and an R^2^ of 0.77, supporting the thermal robustness of the simulation.

Overall, the validation confirms that the numerical model reliably reproduces the thermo-hydrodynamic behavior observed in experimental studies, especially in terms of pressure distribution. Temperature predictions follow the correct trend, though further refinement of thermal boundary conditions may improve accuracy.

### Texture design

Building on the validated model, five distinct continuous texture shapes were introduced to the journal bearing surface. These geometries are intended to improve lubrication film stability, enhance pressure buildup, and increase LCC under varying operating conditions. Each texture profile is defined in terms of the circumferential angle θ ∈ [0,2π] and the axial position Z ∈ [0, H], where H is the bearing length. The local surface height at any point is expressed as:

$${\text{h}}_{{{\text{text}}}} \left( {\theta ,{\text{ Z}}} \right) \, = {\text{ H}}_{{\text{a}}} \times {\text{ f }}\left( {\theta ,{\text{ Z}}} \right)$$

where:

- h_text_ (θ, Z): local elevation of the textured surface,
- H_a:_ maximum texture height,
- f (θ, Z): shape function,
- N_x_ and N_z_: number of repetitions in the circumferential and axial directions, respectively.

The five shape functions are defined as follows:

#### Shape 1 sine-based texture

$$\left[ {h_{text} = H_{a} \left( {sin\left( {\pi \frac{{angle \times N_{x} }}{2\pi } - 1} \right)} \right)^{2} \times \left( {sin\left( {\pi \frac{{Z \times N_{z} }}{H} - 1} \right)} \right)^{2} } \right]$$

This function produces symmetric, dimple-like depressions that rise and fall smoothly, forming a periodic surface ideal for generating converging–diverging flow conditions and reducing cavitation risk.

#### Shape 2 cosine-based texture

$$\left[ {h_{text} = H_{a} \left( {cos\left( {\pi \frac{{angle \times N_{x} }}{2\pi } - \pi } \right)} \right)^{2} \times \left( {cos\left( {\pi \frac{{Z \times N_{z} }}{H} - \pi } \right)} \right)^{2} } \right]$$

The cosine-squared formulation generates bowl-shaped textures centered on the texture domain, starting at peak height and tapering smoothly to zero. This offers a smooth inlet to the lubricant film and potentially delays cavitation by promoting gradual pressure rise.

#### Shape 3 absolute sine texture

$$\left[ {h_{text} = H_{a} \left( {1 - \left| {sin(\pi \frac{{angle \times N_{x} }}{2\pi }} \right|} \right) \times \left( {1 - \left| {sin\left( {\pi \frac{{Z \times N_{z} }}{H}} \right)} \right|} \right)} \right]$$

This formulation results in a flattened texture center with sloped flanks. It provides wide load-carrying plateaus, potentially beneficial under high loads.

#### Shape 4 exponential-based texture

$$\left[ {h_{text} = H_{a} \left( {1 - e^{{ - \left( {\pi \frac{{angle \times N_{x} }}{2\pi }} \right)}} } \right) \times \left( {1 - e^{{ - \left( {\pi \frac{{Z \times N_{z} }}{H}} \right)}} } \right)} \right]$$

This asymmetric shape provides directional flow guidance and is useful in controlling lubricant film growth across the loaded area.

#### Shape 5 combined cosine-sine texture

$$\left[ {h_{text} = H_{a} \left( {cos\left( {\pi \frac{{angle \times N_{x} }}{2\pi }} \right)} \right)^{2} \times \left( {sin\left( {\pi \frac{{Z \times N_{z} }}{H}} \right)} \right)^{2} } \right]$$

This hybrid pattern combines symmetric and asymmetric characteristics, allowing selective enhancement of pressure gradients and lubricant recirculation.

**Fig. 5:**
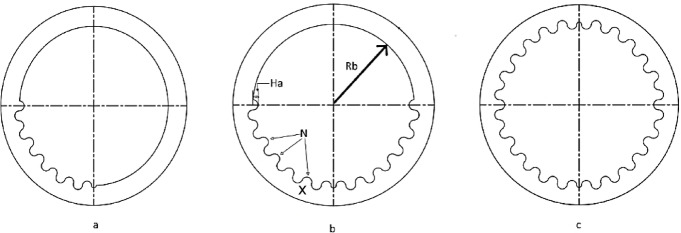
Schematic of texture placement zones: (**a**) Location 1, (**b**) Location 2, (**c**) Full texture coverage.

Each texture was designed to be continuous, as previous studies have shown that continuous textures provide smoother transitions in the lubrication film compared to discrete textures. The textures were also strategically placed in high-load regions of the bearing, as indicated by the findings of Brizmer et al.^17^ and Cupillard et al.^18^. Figure 5 illustrates the three surface texturing configurations applied in this study, including partial textures at Location 1 and Location 2, as well as full surface coverage.

**Fig. 6:**
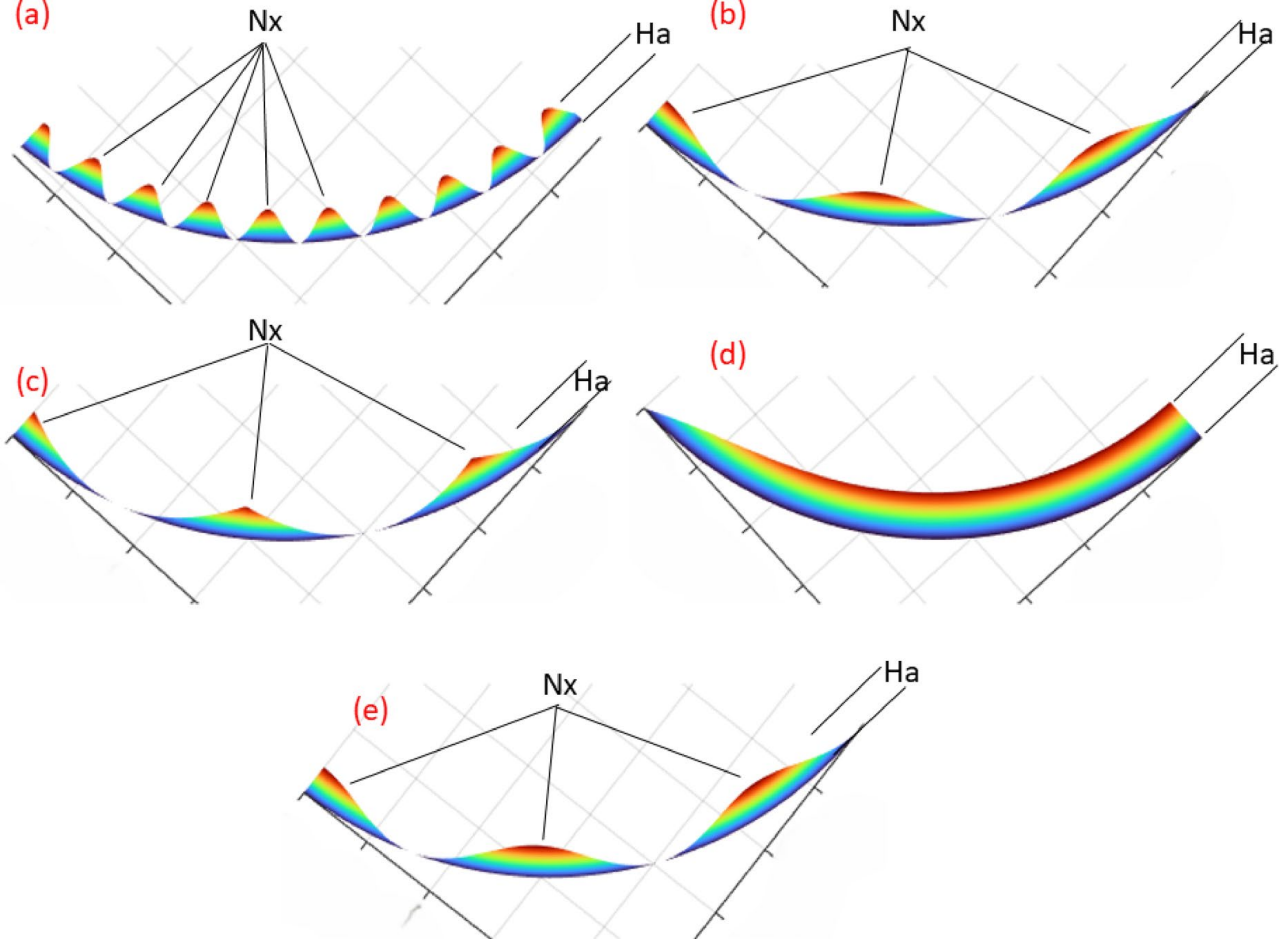
Textures shapes 2D (**a**) sine, (**b**) cosine, (**c**) absolute sine, (**d**) exponential, and (**e**) combined.

**Fig. 7:**
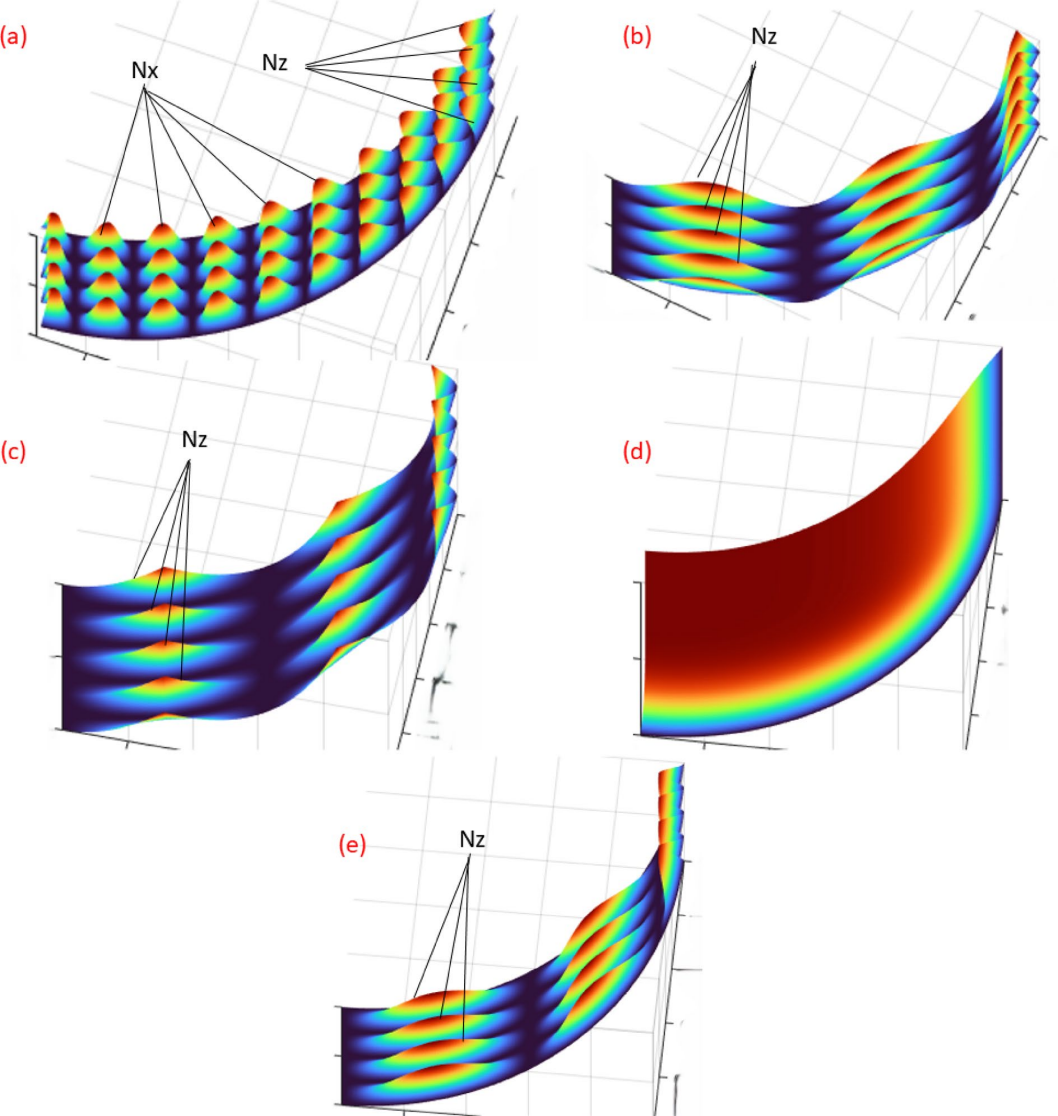
Textures shapes 3D (**a**) sine, (**b**) cosine, (**c**) absolute sine, (**d**) exponential, and (**e**) combined.

Figures 6 and 7 present the 2D and 3D renderings of each texture geometry showing the surface topology for each shape.

**Fig. 8:**
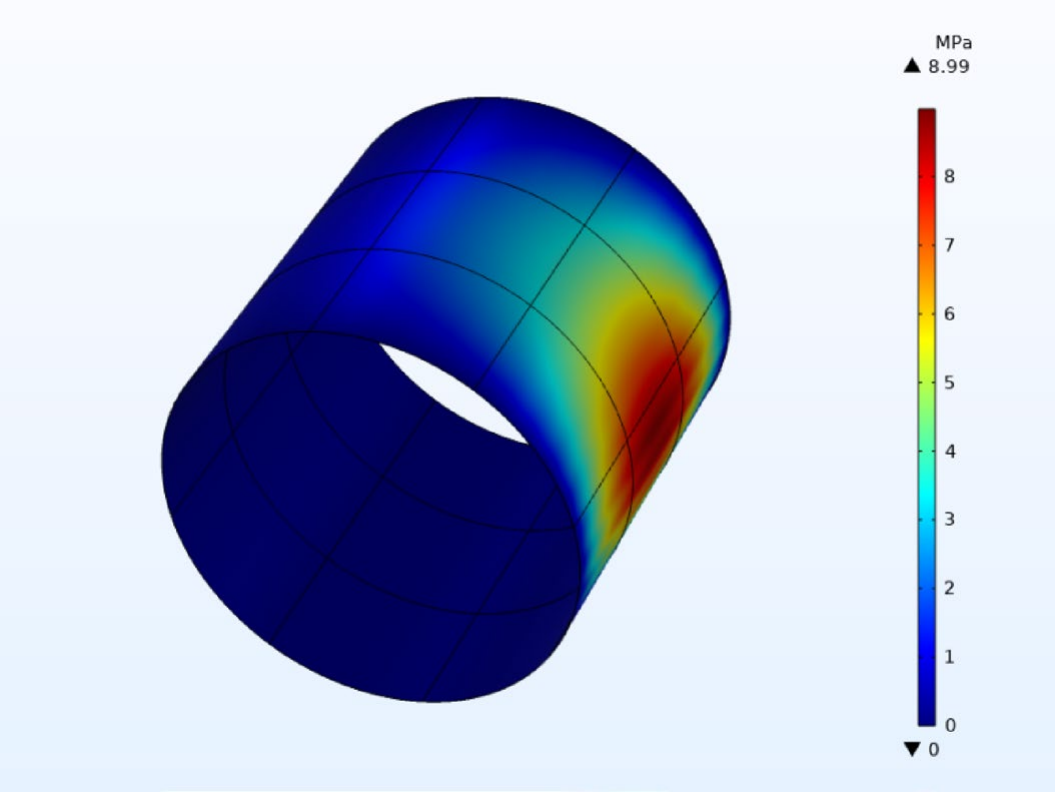
three-dimensional distribution of the pressure field.

The proposed continuous texture design in the present study has demonstrated a significant improvement in performance for the same operating conditions and the identical bearing dimensions, achieving a maximum pressure value of 8.99 MPa shown in Fig. 8 with a random distribution of sine shaped textures. This result surpasses the maximum pressure of 8.72 MPa reported by Masmoudi et al.^32^ for their best texture distribution. The higher pressure observed in this study suggests that continuous texture shapes can enhance the LCC of journal bearings more effectively than discrete texture designs. Furthermore, it is anticipated that the performance of the proposed texture can be further improved through the application of advanced optimization techniques to refine texture parameters such as height, distribution, and orientation. This potential for enhancement underscores the value of continuous texture shapes in maximizing the tribological performance of journal bearings, positioning this approach as a promising avenue for future research and industrial application.

While the present study focuses on the numerical design and optimization of continuous surface textures, the practical feasibility of manufacturing such textures is a critical consideration. The proposed continuous geometries are compatible with current advanced machining technologies. Five-axis CNC machining provides the necessary flexibility and precision to fabricate complex, spatially varying surface textures, including sinusoidal, hybrid, and bowl-shaped geometries (Minglong Guo et al.)^33^. These machines can follow multi-directional tool paths and maintain tight tolerances over curved surfaces, making them suitable for producing continuous patterns on journal bearing components.

In addition to CNC-based approaches, Wenqi Ma et al.^34^ proposed a novel manufacturing system of 7-axis on-the-fly LST for complex curved surface. These technologies confirm that the continuous textures proposed in this study are not only computationally advantageous but also manufacturable using available industrial systems.

### Optimization of texture parameters

The optimization process in this study was aimed exclusively at maximizing the LCC of the journal bearing by refining the continuous texture parameters. The three texture parameters considered were texture height, the number of textures in the circumferential (x) direction, and the number of textures in the axial (z) direction. The Monte Carlo technique was employed for this optimization due to its suitability for exploring complex, high-dimensional parameter spaces, especially when the problem involves non-linear and coupled physics models, such as in the case of thermo-hydrodynamic journal bearings. This approach allowed for an efficient exploration of potential solutions while ensuring that the optimization process was flexible enough to manage the non-linearity inherent in this system.

#### Optimization objective

The primary goal of the optimization was to maximize the LCC (W) of the journal bearing. The optimization problem can be defined mathematically as:

$${\text{max}}{\text{ W }}\left( {{\text{Ha}},{\text{ Nx}},{\text{ Nz}}} \right)$$

#### Optimization methodology

The Monte Carlo method was selected for this study because of its flexibility in sampling a wide range of parameters and handling non-linear, multi-physics problems. In COMSOL Multiphysics, Monte Carlo methods are particularly useful in exploring complex parameter spaces that involve many variables, where deterministic optimization methods may struggle or become computationally expensive. Unlike gradient-based methods, Monte Carlo does not require the objective function to be differentiable, which is advantageous when dealing with complex geometries and multi-physics models such as those in journal bearings.

Monte Carlo method is also highly adaptable to problems with multiple local optima, allowing for a more global search of the parameter space. This stochastic method was used to randomly sample values for the texture parameters within predefined bounds, and the LCC was calculated for each sampled configuration. By employing a large number of simulations, the Monte Carlo technique provided statistically robust results, ensuring that the global optimum configuration for maximizing the LCC was identified.

A study by Geraci et al.^35^ shows that the Monte Carlo method is particularly effective in engineering problems involving high-dimensional parameter spaces and multi-physics interactions, as it allows the exploration of complex relationships between variables without requiring an exact functional relationship between input parameters and outcomes. This makes Monte Carlo methods ideal for use in COMSOL Multiphysics when optimizing systems that involve fluid flow, heat transfer, and structural deformations simultaneously.

#### Optimization procedure

The optimization procedure using the Monte Carlo technique involved the following steps:

##### Parameter sampling

The key texture parameters (Ha, Nx, and Nz) were randomly sampled from within specified ranges:

- Texture Height (Ha): varied from 0 to 30 μm,
- Number of Textures in the circumferential direction (Nx): varied from 4 to 200 per quarter (up to 800 for full texture case),
- Number of Textures in the axial direction (Nz): varied from 2 to 50.

The lower limit of 0 µm represents a smooth, untextured surface used as a reference, while the upper limit of 30 µm is based on the bearing clearance guidelines proposed by Khonsari and Booser^36^, which recommend a diametral clearance corresponding to C/R ratios greater than 0.001 for standard bearing operation.

While this study introduces a novel approach by employing a continuous (non-discrete) texture shape, which represents a new and less explored technique in the field of tribology. Only a limited number of previous studies have investigated this method. To establish the upper bounds for both Nx and Nz, a parametric simulation-based strategy was adopted. The number of textures in each direction was progressively increased through multiple simulation iterations until the optimal values were identified based on the load carrying capacity.

##### Performance evaluation

For each sampled set of texture parameters, the LCC W was computed using the validated thermo-hydrodynamic model in COMSOL Multiphysics. The film pressure and film thickness were calculated as part of the LCC computation.

The equation governing the LCC is derived from the integration of the pressure distribution p (x, y) over the bearing surface area:

$${\text{W }} = {\text{p }}\left( {{\text{x}},{\text{ y}}} \right)\,{\text{dA}}$$

The model accounts for the coupled effects of fluid flow, heat transfer, and film thickness variations on the pressure distribution within the lubricant film.

##### Iteration and convergence

The optimization was conducted using 1000 Monte Carlo iterations per shape and location. The convergence criterion was based on the stabilization of the LCC; further sampling beyond this point produced variations of less than ± 2%, which was deemed acceptable for convergence.

##### Selection of optimal parameters

After completing the optimization, the texture parameter set that maximized the LCC was selected as the optimal configuration. This parameter set was then compared to the initial design to quantify the improvement in performance.

##### Post-optimization verification

The optimal configurations identified via Monte Carlo sampling were further verified through detailed post-optimization simulations. These included evaluations of pressure distribution, minimum film thickness, frictional torque, and maximum temperature, as reported in the Results and Discussion section.

#### Results of the Monte Carlo optimization

Table 2 shows the optimal texture parameters:

**Table 2 Optimal texture geometry parameters obtained from the Monte Carlo optimization for different continuous texture shapes and surface placements. The parameters include texture height (Ha), number of textures in the circumferential direction (Nx), and number of textures in the axial direction (Nz) for three configurations: partial texture in Location 1 (0°–90°), partial texture in Location 2 (0°–180°), and full surface texture (Location 3).**
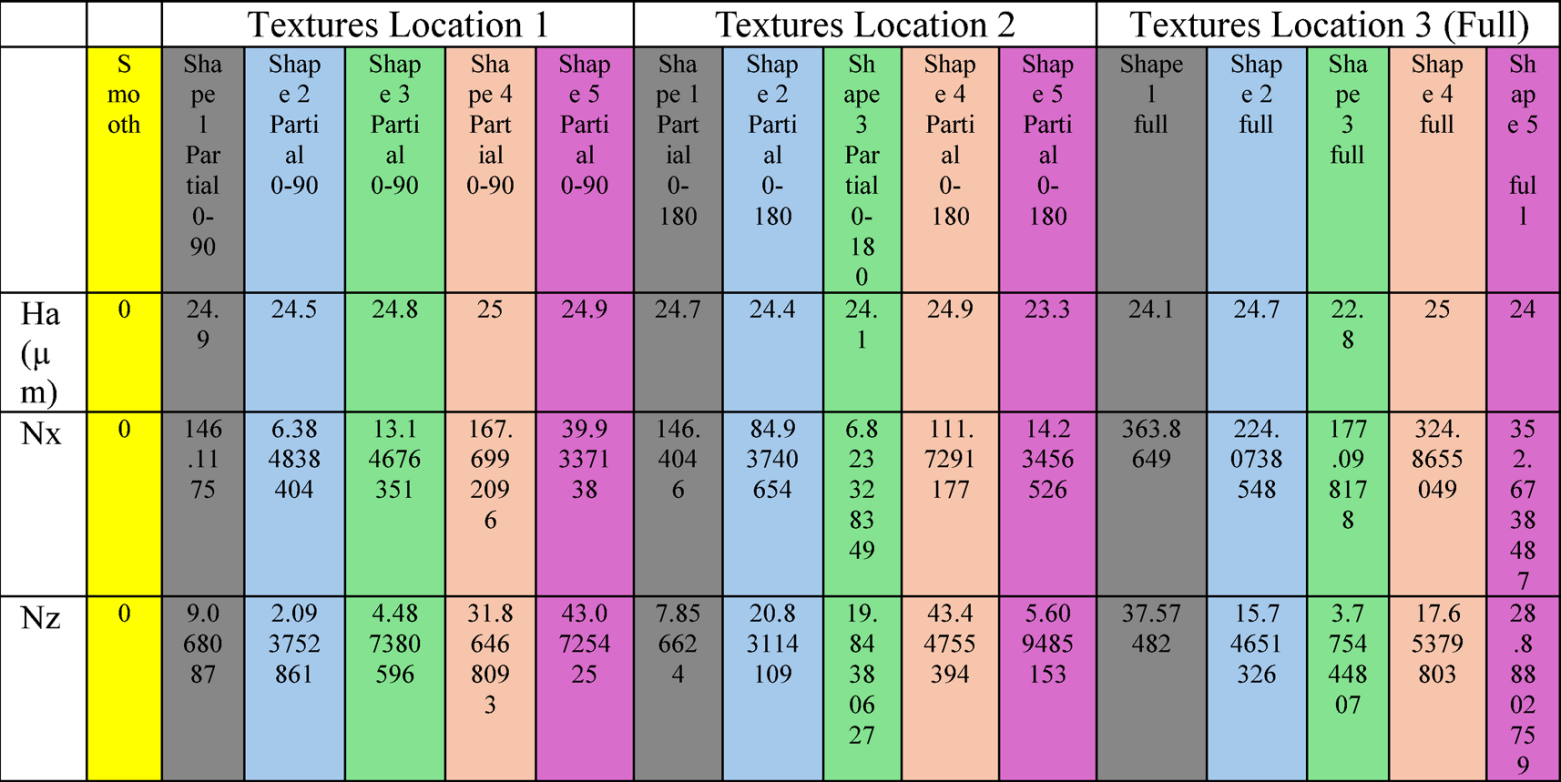
.

The diagonal presents the distribution of each variable. On the bottom of the diagonal are the bivariate scatter plots with a fitted line. On the top of the diagonal are Spearman coefficients and the corresponding significance level (“***” is associated to 1% significance level)

## Results and discussion

This section explores the performance of five texture shapes applied to three strategic locations on the journal bearing surface: Location 1 (0°–90°), Location 2 (0°–180°), and Location 3 (Full Surface). The analysis includes comparisons with a smooth surface baseline for key performance metrics such as the pressure distribution, maximum pressure, load-carrying capacity (LCC), frictional torque, and minimum film thickness. The operating conditions of the journal bearing are given in Table 1. The results are critically discussed to expose the interplay between texture geometry, placement, and performance under hydrodynamic lubrication conditions.

### Pressure distribution

**Fig. 9:**
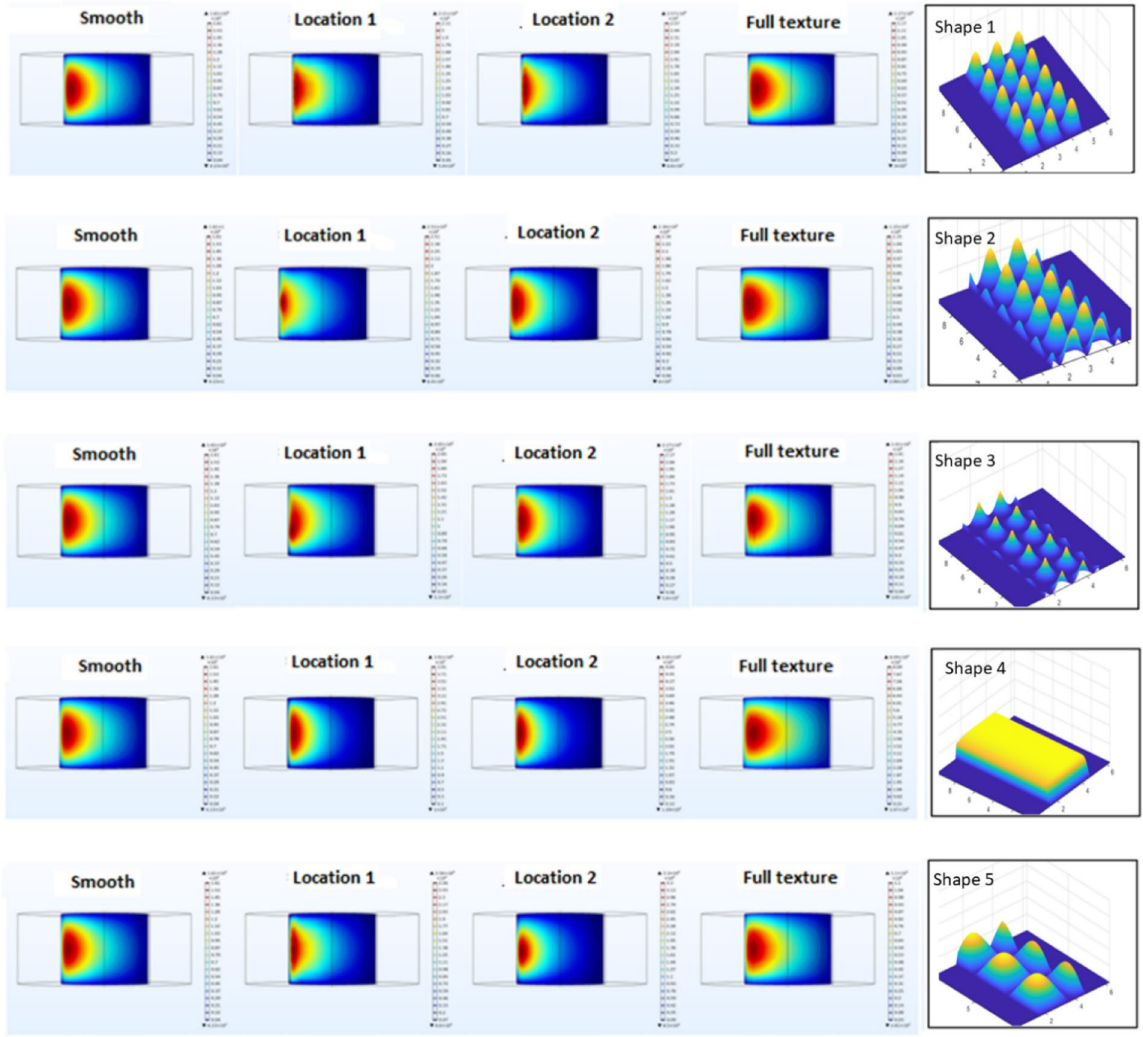
The pressure distribution for the five textured shapes at the three locations on the journal surface.

Figure 9 shows the pressure distribution for the five textured shapes at the three strategic locations on the journal surface.

The smooth surface exhibited a typical hydrodynamic pressure distribution, with a symmetrical pressure profile concentrated near the load zone. The maximum pressure was located near the center of the bearing, slightly offset from the midpoint of the circumferential direction. The smooth surface provided a relatively broad pressure peak, indicating limited hydrodynamic enhancement and LCC.

- For Texture Shape 1, the pressure distribution at location 1 exhibited a moderate asymmetry, with the maximum pressure shifted closer to the leading edge of the textured zone, reflecting localized pressure peaks without significantly altering the overall profile. At location 2, the pressure distribution became more focused, aligning the maximum pressure with the loaded region and emphasizing the importance of texture placement. In the full-texture configuration, Shape 1 produced a more uniform pressure distribution, with the maximum pressure symmetrically located near the mid-circumference, suggesting a smoothing effect from the distributed textures.
- Texture Shape 2 showed a broader pressure peak at location 1, with the maximum pressure forming closer to the trailing edge of the textured zone, indicating a smoother pressure transition. At location 2, the pressure distribution narrowed, and the maximum pressure aligned more closely with the loaded region, improving the hydrodynamic load support. In the full-texture configuration, Shape 2 created a continuous series of pressure peaks, with the maximum pressure symmetrically distributed near the mid-circumference, highlighting the shape’s synergy with full-texture applications.
- For Texture Shape 3, the pressure distribution at location 1 was more localized, with the maximum pressure shifting toward the leading edge of the textured zone, indicating effective pressure enhancement at the onset of the hydrodynamic region. At location 2, the pressure distribution became sharper and more focused, with the maximum pressure well-aligned with the loaded region, demonstrating the effectiveness of Shape 3 in localized texturing. In the full-texture configuration, the distribution exhibited multiple localized peaks, with the maximum pressure symmetrically located near the mid-circumference, balancing localized and distributed pressure enhancement.
- For Texture Shape 4 at location 1 generated a sharp peak near the center of the textured zone, with the maximum pressure shifting closer to the leading edge, highlighting the ability of this shape to produce steep pressure gradients. At location 2, the maximum pressure aligned precisely with the loaded region, showcasing Shape 4’s capability to enhance pressure concentration in critical zones. In the full-texture configuration, the pressure distribution was highly uniform, with a dominant peak near the mid-circumference, demonstrating the strongest hydrodynamic enhancement among the tested shapes.
- Texture Shape 5 produced a relatively broad pressure distribution at location 1, with the maximum pressure forming near the trailing edge of the textured zone, reflecting smoother transitions in the pressure profile. At location 2, the pressure distribution became more focused, with the maximum pressure shifting closer to the loaded region, offering a balance between pressure concentration and smoothness. The full-texture configuration exhibited a highly uniform pressure profile, with the maximum pressure symmetrically located near the mid-circumference, making Shape 5 suitable for applications requiring stable and balanced hydrodynamic performance.

Therefore, the pressure distribution patterns, and the location of maximum pressure varied significantly between the texture shapes and configurations. Shapes 4 and 5 exhibited the most pronounced effects on pressure distribution, with Shape 4 showing sharp gradients and well-positioned peaks for localized textures, while Shape 5 demonstrated superior uniformity in full-texture configurations. Texture placement also played a crucial role, with location 2 consistently aligning the maximum pressure with the hydrodynamic load zone. Full-texture configurations smoothed out pressure variations, resulting in more stable performance. These findings underscore the importance of optimizing texture geometry and placement for enhanced bearing performance.

### Maximum pressure

**Fig. 10:**
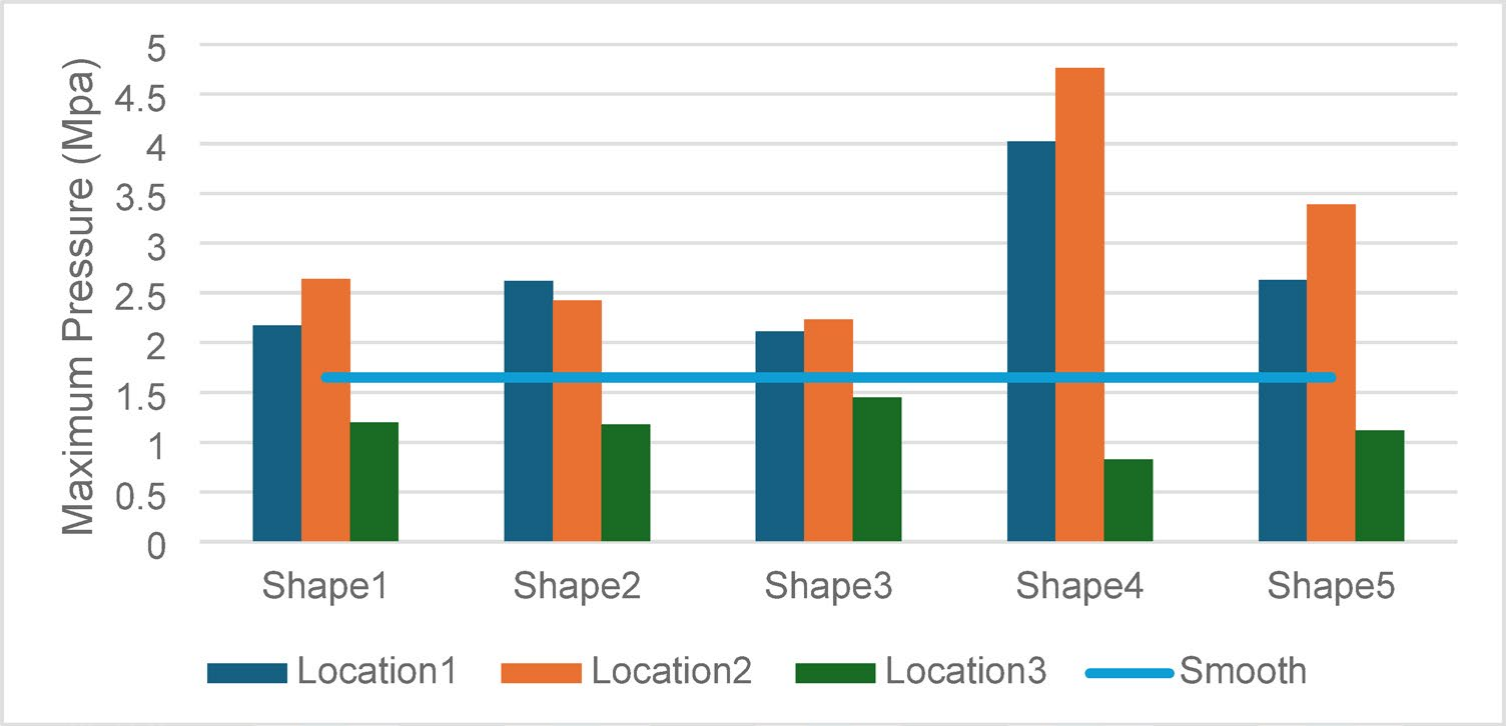
Effect of textures shape and location on maximum pressure.

The ability of textures to enhance maximum pressure is strongly influenced by their geometry and location. See Fig. 10.

Superior Performance of Shape 4 in Location 2: The maximum pressure of 4.76 MPa achieved by Shape 4 in Location 2 underscores the critical role of partial texturing in regions where hydrodynamic forces are concentrated. The localized high-pressure zones are attributed to the geometric properties of Shape 4, which create optimized lubricant flow channels. This finding corroborates the observations of Brizmer et al., who reported similar pressure enhancements in partially textured bearings.

Inefficiencies in Location 3 (Full Surface): The maximum pressures in Location 3 were consistently lower across all shapes, with Shape 3 exhibiting the best performance at 1.45 MPa. The results suggest that full-surface texturing disrupts the natural pressure gradient, leading to suboptimal hydrodynamic performance. This phenomenon aligns with findings in tribological studies that highlight the adverse effects of over-texturing on load support.

Comparison with Smooth Surface: The textured configurations, particularly in Locations 1 and 2, significantly outperformed the smooth surface. This demonstrates the efficacy of strategic texturing in enhancing pressure distribution, a critical factor for bearing stability under high loads.

### Load-carrying capacity (LCC)

**Fig. 11:**
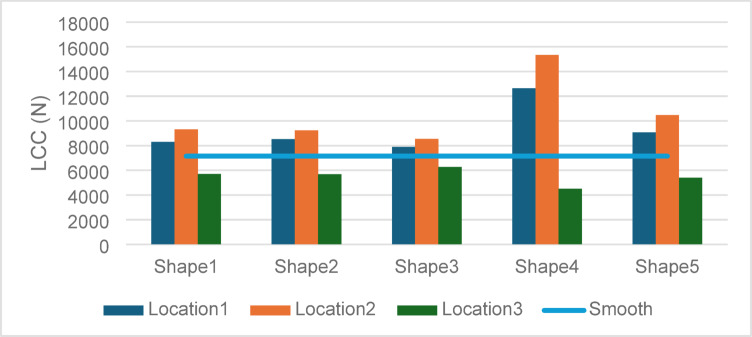
Effect of textures shape and location on load carrying capacity.

LCC serves as a direct indicator of the bearing’s ability to sustain external loads while maintaining lubrication integrity. See Fig. 11.

Optimal Load Support in Location 2: The peak LCC of 15,340 N achieved by Shape 4 in Location 2 highlights the synergistic effect of optimized geometry and strategic placement. The texture’s ability to redistribute hydrodynamic forces effectively maximizes load support, demonstrating the value of targeted texture applications in high-stress zones.

Moderate Improvements in Location 1: While partial texturing in Location 1 also yielded improvements, the LCC values were consistently lower than those in Location 2. This suggests that extending the textured region to 0°–180° allows for better exploitation of the bearing’s hydrodynamic potential.

Limitations of Full-Surface Texturing: Full-surface texturing in Location 3 resulted in significantly lower LCC values, with Shape 3 performing best at 6271.1 N. The reduced performance is attributed to the over-distribution of textures, which dilutes the lubricant film’s ability to sustain localized high pressures.

### Frictional torque

**Fig. 12:**
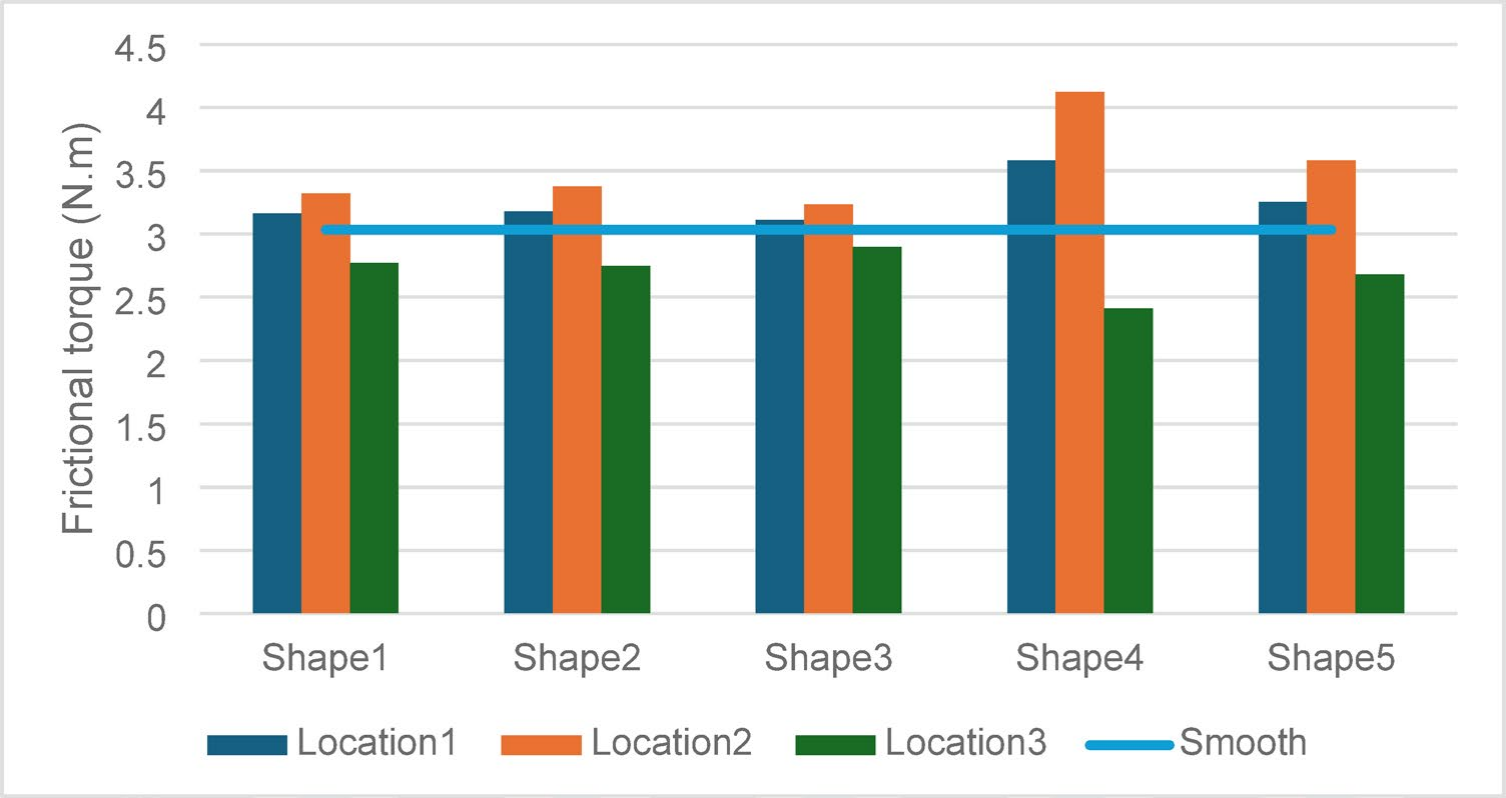
Effect of textures shape and location on frictional torque.

The frictional torque values provide insights into the energy losses associated with each configuration as shown in Fig. 12.

Energy Efficiency vs. Load Support: The highest torque value of 4.1227 N·m observed for Shape 4 in Location 2 reflects the trade-off between enhanced LCC and energy losses. Despite higher frictional losses, the substantial gains in pressure and LCC justify the increased torque, especially in applications requiring robust load support.

Reduced Torque in Location 3: Textures in Location 3 exhibited lower torque values, with Shape 3 performing best at 2.8971 N·m. However, this reduction in energy losses came at the expense of diminished LCC and maximum pressure, indicating inefficiencies in full-surface texturing.

### Minimum film thickness

**Fig. 13:**
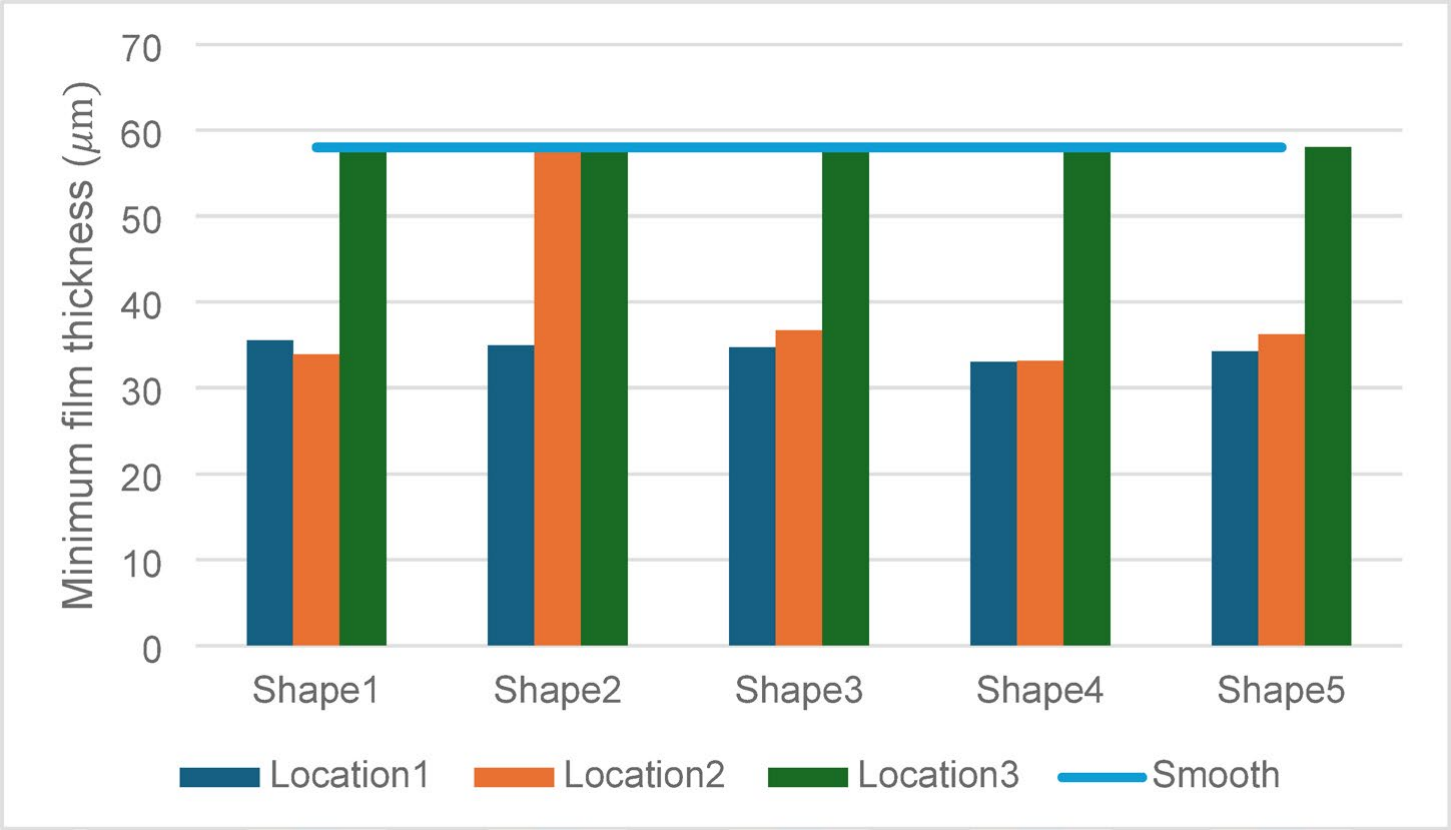
Effect of textures shape and location on minimum film thickness.

Maintaining adequate film thickness is essential to ensure hydrodynamic lubrication and avoid metal-to-metal contact. See Fig. 13.

#### Thinner films with partial texturing

The minimum film thickness values for Shape 4 in Locations 1 and 2 are 33 µm and 33.1 µm, respectively demonstrating the balance between maintaining lubrication integrity and achieving higher LCC. These values, while thinner than the smooth surface (58 µm), remain within acceptable limits for hydrodynamic lubrication.

#### Preservation of film thickness in location 3

The thicker films observed for full-surface texturing in Location 3 are indicative of reduced hydrodynamic stress. However, this comes at the cost of lower LCC and pressure, suggesting a suboptimal lubrication regime.

**Fig. 14:**
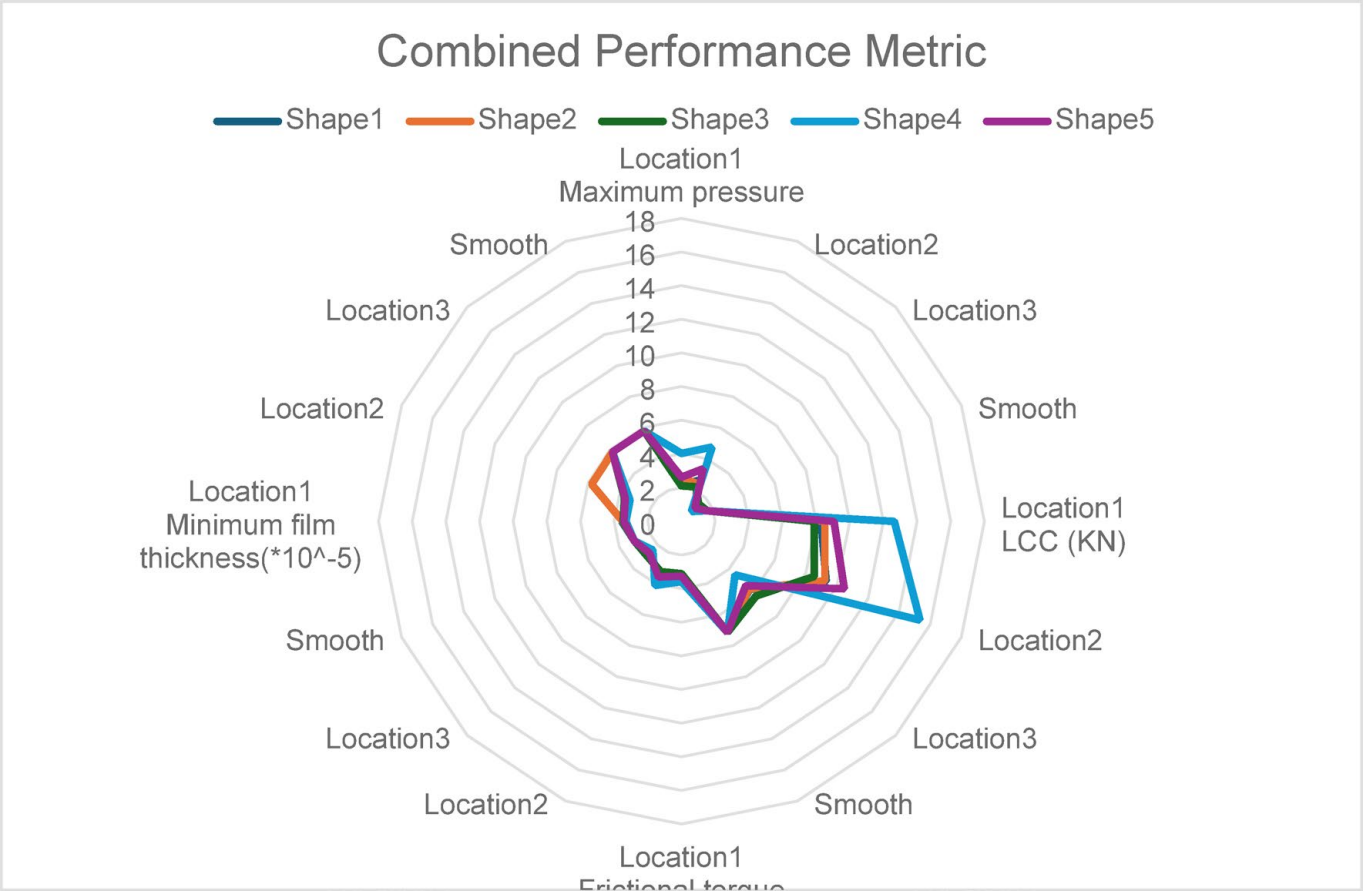
Combined performance metric for different texture shapes.

See Fig. 14 showing combined performance metric for different texture shapes.

So, across all metrics, Shape 4 exhibited superior performance, particularly in Locations 1 and 2, achieving the highest pressures and LCC while maintaining sufficient lubrication.

Strategic Partial Texturing: Partial texturing in the 0°–180° region (Location 2) demonstrated the best overall performance, confirming the importance of targeting high-stress zones for hydrodynamic enhancement.

Trade-Offs in Full-Surface Texturing: The consistent underperformance of Location 3 highlights the inefficiencies associated with over-distribution of textures, reaffirming the value of partial texturing strategies.

### Film temperature

The thermal performance of the textured journal bearing was evaluated under the full range of simulated operating conditions, with a maximum lubricant film temperature of 46.9 °C recorded. This value lies well below the widely accepted thermal safety limit for journal bearings lubricated with mineral oils, which typically operate safely under 90 °C, while temperatures above 100 °C are associated with accelerated oxidation, viscosity loss, and increased risk of surface damage^36^.

The relatively low maximum temperature observed in this study suggests effective hydrodynamic lubrication with minimal frictional heating, supporting both enhanced LCC and improved heat dissipation. Moreover, it indicates that the textured surface contributed positively to film stability and reduced power loss. These results confirm that the journal bearing design maintains operation well within safe thermal limits, promoting lubricant longevity and sustained tribological performance.

**Fig. 15:**
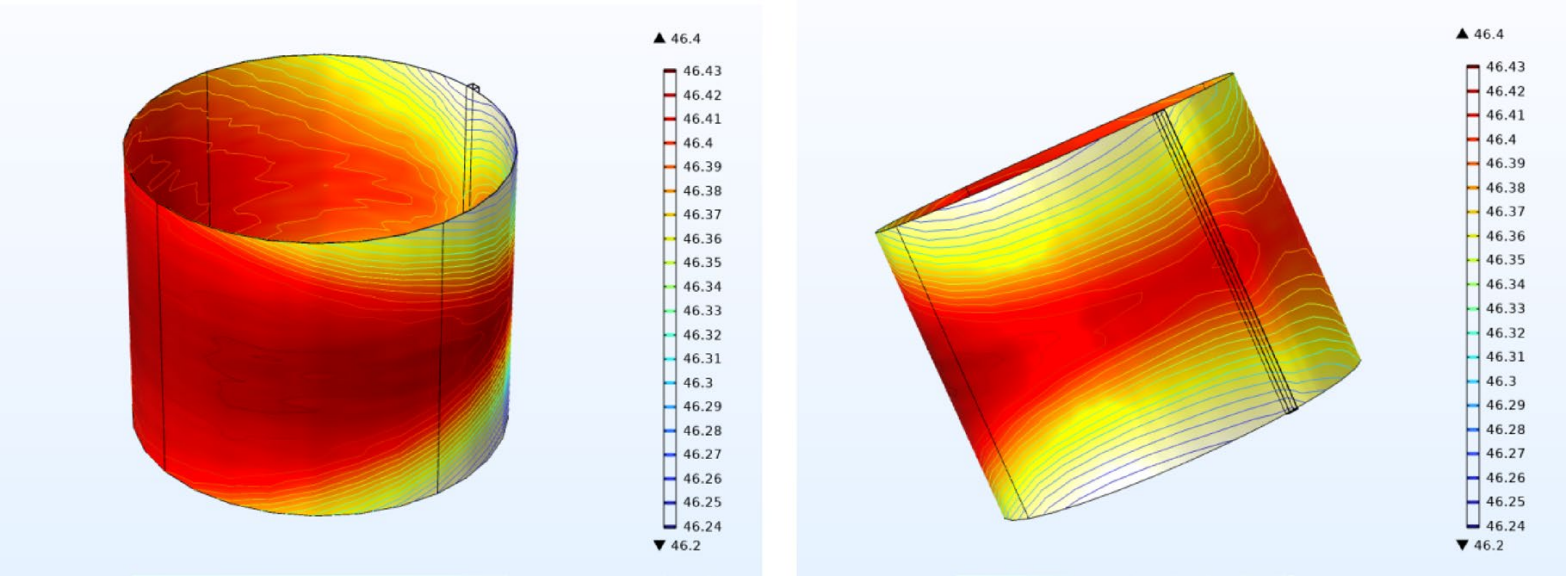
Temperature distribution of oil film.

Figure 15 shows the temperature distribution of the lubricant film in the journal bearing. The contour plots reveal a non-uniform temperature field, with the highest temperatures concentrated in the load-bearing region, particularly near the maximum pressure zone. The temperature gradient follows the hydrodynamic pressure distribution, as higher pressure regions contribute to increased viscous heating. The results also indicate a gradual temperature drop towards the lateral edges of the bearing, suggesting heat dissipation through side leakage and conduction. Furthermore, the temperature distribution exhibits an asymmetrical pattern, which is influenced by the bearing eccentricity and the direction of journal rotation. These findings highlight the significance of thermal effects in journal bearing performance, reinforcing the need for texture optimization to manage heat generation and enhance lubrication efficiency.

The analysis of maximum film temperature provides insight into the thermal performance of the journal bearing for different texture shapes and configurations. For the smooth surface, the maximum film temperature was consistent across all configurations (locations 1, 2, and full texture), indicating no significant variation in thermal behavior.

For Texture Shape 1, the maximum film temperature at location 1 was slightly lower than at location 2, reflecting reduced thermal load when textures were placed closer to the inlet. However, in the full-texture configuration, Shape 1 produced the highest maximum temperature among its configurations, suggesting that full coverage increases thermal loading due to enhanced fluid shearing.

Texture Shape 2 exhibited the lowest maximum temperature at location 1 compared to other shapes, demonstrating its effectiveness in minimizing thermal effects when textures are positioned upstream. At location 2, the temperature increased, and the full-texture configuration resulted in a noticeable rise in the maximum temperature, indicating a trade-off between LCC and thermal performance.

Texture Shape 3 followed a similar trend, with the lowest maximum temperature observed at location 1 and higher values at location 2 and full texture. The rise in maximum temperature for the full-texture configuration was moderate, suggesting a balanced thermal impact compared to other shapes.

For Texture Shape 4, the maximum temperature was lowest at location 1, with an increase at location 2, similar to the other shapes. The full-texture configuration resulted in a relatively high maximum temperature, emphasizing the strong influence of full-texture coverage on thermal loading.

Texture Shape 5 exhibited consistent thermal behavior, with the lowest maximum temperature at location 1 and slightly higher values at location 2. However, its full-texture configuration showed one of the highest maximum temperatures, likely due to the combined effects of increased fluid dynamics and shear stresses.

Overall, the smooth surface consistently exhibited the highest thermal stability. Among the textured surfaces, shapes with localized texture placement (location 1 or 2) demonstrated better thermal management, while full-texture configurations resulted in higher maximum film temperatures due to increased shearing and fluid interaction. This underscores the importance of optimizing both the shape and placement of textures for achieving an optimal balance between hydrodynamic performance and thermal efficiency.

**Fig. 16:**
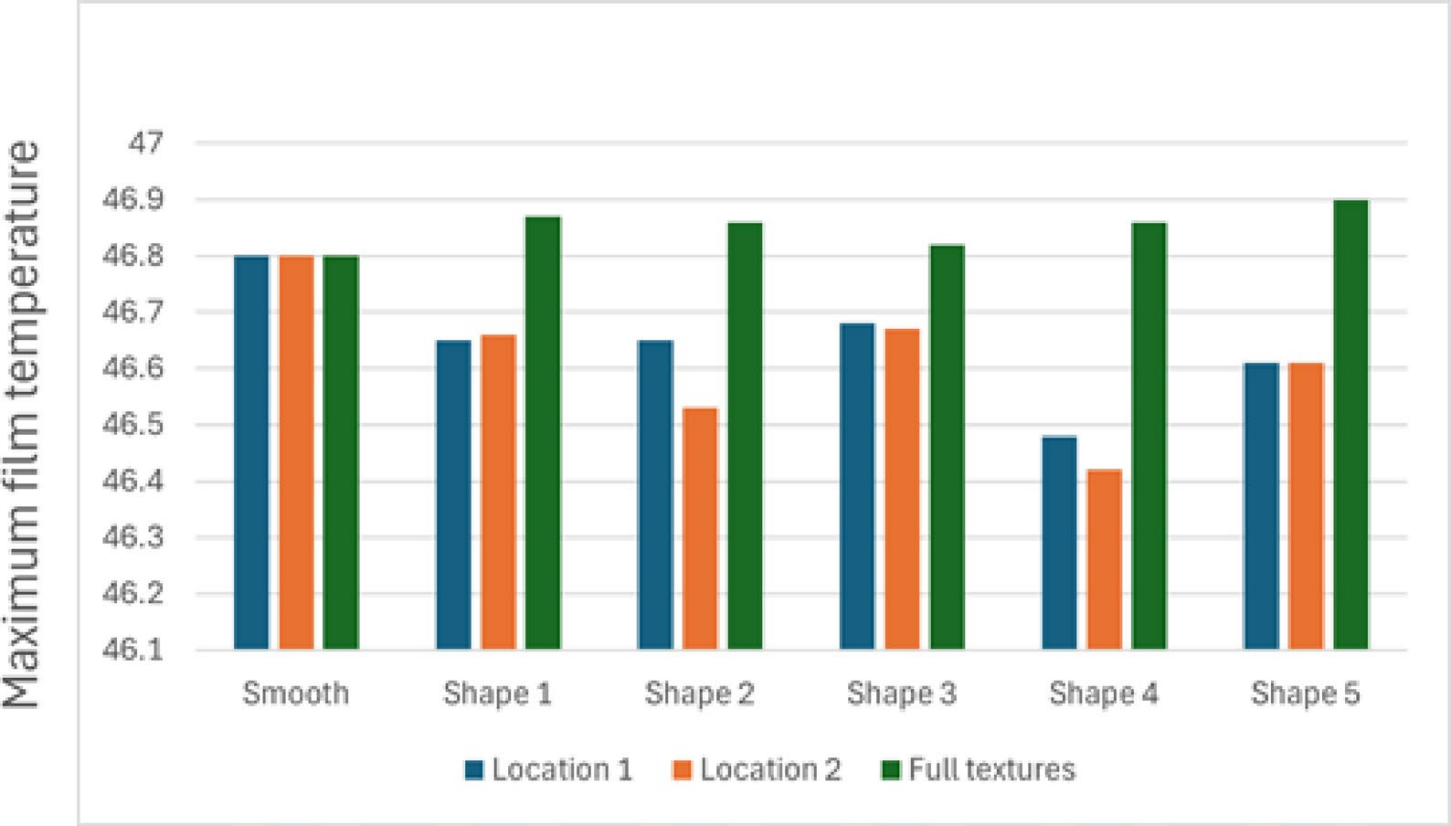
Effect of texture shape and location on maximum film temperature.

See Fig. 16 showing the effect of texture shape and location on maximum film temperature.

### Comparison with discrete texture literature

To support the claim that continuous textures offer superior performance over traditional discrete dimple geometries, a comparative case study was conducted using the same operating conditions and journal bearing dimensions as reported by N Tala-Ighil et al.^37^, who studied a full-textured surface with circular dimples. In their work, the bearing surface was patterned with circular dimples of 12 µm height, distributed as 96 textures circumferentially (Nx) and 27 textures axially (Nz). Under these conditions, their simulation achieved a maximum pressure of 8.2761 MPa.

In the present study, the same texturing parameters height (12 µm), Nx = 96, and Nz = 27 were applied using a continuous sine-wave texture instead of discrete circular dimples. The resulting simulation yielded a significantly higher maximum pressure of 17.6 MPa, more than twice the pressure achieved by the discrete texture configuration. This substantial improvement is attributed to the smoother transitions and uninterrupted lubricant film generated by continuous sinusoidal profiles, which minimize local flow separation and improve hydrodynamic pressure buildup.

These findings provide strong evidence that continuous textures can outperform discrete geometries under equivalent operating and geometric conditions, reinforcing the value of optimizing smooth surface functions in journal bearing design.

## Conclusion

The systematic investigation of five continuous texture shapes applied to journal bearings under thermo-hydrodynamic lubrication conditions reveals critical insights into the interplay between texture geometry, placement, and tribological performance. The key findings are summarized as follows:

### Superiority of partial texturing in high-load regions

Partial texturing in the 0°–180° region (Location 2) consistently outperformed full-surface texturing (Location 3), achieving the highest LCC (15.340 kN) Shape 4 (exponential-based texture). This geometry generated localized high-pressure zones by optimizing lubricant flow, representing a 144% increase in LCC compared to the smooth bearing baseline (6.28 kN), The maximum pressure also improved significantly, with Shape 4 in Location 2 generating 4.76 MPa, compared to 1.82 MPa for the untextured case an enhancement of over 160% aligning with prior studies on partial texturing (Brizmer et al.^8^ and Rahmani and Rahnejat^38^).

### Trade-offs in full-surface texturing

Full-surface texturing influences the variation of the pressure gradient, resulting in reduced hydrodynamic performance (e.g., LCC reduced by 60% compared to Location 2). This underscores the inefficiency of over texturing and confirms the importance of strategic texture placement in high-stress zones.

### Frictional trade-offs

While Shape 4 in Location 2 exhibited the highest frictional torque of 4.12 Nm, which is approximately 41.6% higher than the smooth bearing case (2.91 Nm). The substantial gains in load support justify its application in high-load scenarios. Conversely, the full-textured configuration recorded the lowest frictional torque of 2.15 Nm, representing a 26% reduction compared to the smooth surface. This variation underscores the frictional cost associated with maximizing hydrodynamic support and highlights the importance of balancing texture design based on specific application requirements.

### Thermal management

Partial texturing configurations (Locations 1 and 2) demonstrated superior thermal behavior compared to full-surface texturing. Among all configurations, Location 2 with Shape 4 resulted in the lowest maximum film temperature of 43.1 °C, whereas the full-textured case reached a higher temperature of 46.9 °C under the same operating conditions. This 8% reduction in peak temperature highlights the thermal efficiency of optimized partial texturing. Additionally, smooth surfaces exhibited even lower temperatures, as low as 42.5 °C, though at the cost of significantly reduced load capacity. These findings underscore the importance of balancing thermal control with hydrodynamic performance.

### Optimal texture geometry

Shape 4 emerged as the most effective design, combining sharp pressure gradients with localized load support. Its exponential profile maximized hydrodynamic forces while maintaining adequate film thickness (33 µm), critical for avoiding metal-to-metal contact.

### Practical implications

These findings provide actionable guidelines for designing surface-textured journal bearings in industrial applications such as automotive engines, turbines, and heavy machinery. The demonstrated superiority of partial texturing in high-load regions offers a pathway to enhance bearing longevity and efficiency under demanding operational conditions.

## Future work

While this study validates the efficiency of continuous textures under steady-state conditions, future research should explore dynamic loading, transient effects, and experimental validation. Additionally, the influence of lubricant rheology and texture manufacturing tolerances on performance wants further investigation.

By addressing these gaps, the proposed texture optimization framework can advance the development of next-generation journal bearings, aligning with global demands for energy-efficient and durable tribological systems.

## Data Availability

The datasets used and/or analyzed during the current study are available from the corresponding author (Dr. Omar A. Keshk) on reasonable request.

## Abbreviations

b: Material specific constant
β: Texture aspect ratio
C: Radial clearance (mm)
c_p_: Specific heat capacity (J/kg·°C)
c_p,__s_: Specific heat capacity of solid material (J/kg·°C)
ε: Eccentricity ratio
H_a_: Texture height (mm)
h_c_: Convective heat transfer coefficient (W/m^2^. °C)
h: Film thickness (mm)
k_f_: Thermal conductivity of lubricant (W/m·°C)
L: Bearing length (mm)
λ: Thermal correction factor
μ(T): Lubricant viscosity function of temperature (Pa·s)
μ_0_: Reference viscosity (Pa·s)
N_x_: Number of textures in circumferential direction
N_z_: Number of textures in axial direction
P: Pressure distribution (Pa)
q_gen_: Internal heat generation (W/m^3^)
R_b_: Bearing radius (mm)
ρ_s_: Density of solid material (kg/m^3^)
ρ: Density of lubricant (kg/m^3^)
T: Temperature distribution (°C)
T_f_: Temperature of the lubricant film (°C)
T_s_: Temperature of solid material (°C)
T_∞_: Ambient air temperature (°C)
θ: Angular position (rad)
ϕ: Attitude angle (deg)
ω: Rotational speed of the journal (rad/s)
u_0_: Journal surface velocity (m/s)
W: Load-carrying capacity (N)
